# The Association Between Maladaptive Daydreaming and Attention-Deficit/Hyperactivity Disorder Among Rwandan Medical Students, 2025–2026, Kigali, Rwanda

**DOI:** 10.1192/bjo.2026.11215

**Published:** 2026-06-30

**Authors:** Nida Bakri Elhaj, Ruth Tsigebrhan, Danya Ibrahim

**Affiliations:** 1 University of Medical Sciences and Technology, Khartoum, Sudan; 2 University of Global Health Equity (UGHE), Butaro, Rwanda; 3 University of Khartoum, Khartoum, Sudan

## Abstract

**Aims:**

Maladaptive daydreaming (MD) is a pattern of immersive and emotionally charged daydreaming that can significantly interfere with an individual’s social functioning and daily activities. MD is not formally recognised as a mental disorder in the International Classification of Diseases (ICD) or the Diagnostic and Statistical Manual of Mental Disorders (DSM–5–TR). This study aimed to determine the prevalence of MD and attention-deficit/hyperactivity disorder (ADHD) among medical students, identify the association of the two conditions, and assess the impact of socio-demographic factors.

**Methods:**

A cross-sectional analytical study was conducted among medical students enrolled at the University of Rwanda and the University of Medical Sciences and Technology, with participants primarily residing in Rwanda, Tanzania, and other countries. Respondents were classified into two groups–maladaptive daydreamers (MDers) and non-maladaptive daydreamers (non-MDers)–based on the 16-item Maladaptive Daydreaming Scale (MDS-16). Data were collected using validated paper-based and online Google Forms self-administered questionnaires (MDS-16 and Adult ADHD Self-Report Scale [ASRS-v1.1]). Ethical approval was obtained, and data were analysed using Statistical Package for the Social Sciences (SPSS) version 23.

**Results:**

Our study included 584 respondents (52.9% male, 47.1% female) with a mean age of 21.6 ± 1.7 years. The prevalence of probable MD was 22.9%, while ADHD was reported in 21.4% of participants. Among students with MD, 48% screened positive for ADHD,compared with 16.2% who did not meet criteria for ADHD. Both MD and ADHD were significantly associated with poor academic performance (p <0.003 and p <0.001, respectively). Using logistic regression, there was a strong positive correlation between MD and ADHD (p <0.001; R²=0.168).

**Conclusion:**

MD and ADHD show a significant positive relationship, and their co-occurrence is associated with a measurable decline in academic performance as perceived by the participants. These findings underscore the need for early screening strategies, the provision of counselling and psycho-educational support for affected students. Future longitudinal and randomised studies are warranted to establish standardised diagnostic criteria and therapeutic guidelines for MD and to differentiate it from related attentional and behavioural disorders across diverse populations.

